# Malaria-like symptoms associated with a natural *Plasmodium reichenowi* infection in a chimpanzee

**DOI:** 10.1186/s12936-015-0743-y

**Published:** 2015-05-28

**Authors:** Anaïs Herbert, Larson Boundenga, Anne Meyer, Diamella Nancy Moukodoum, Alain Prince Okouga, Céline Arnathau, Patrick Durand, Julie Magnus, Barthélémy Ngoubangoye, Eric Willaume, Cheikh Tidiane Ba, Virginie Rougeron, François Renaud, Benjamin Ollomo, Franck Prugnolle

**Affiliations:** Centre de Primatologie, Centre International de Recherches Médicales de Franceville, BP 769 Franceville, Gabon; Unité de Biodiversité, Ecologie et Evolution des Parasites (UBEEP), Centre International de Recherches Médicales de Franceville, BP 769 Franceville, Gabon; Société d’Exploitation du Parc de la Lékédi, Bakoumba, Gabon; Laboratoire MIVEGEC; UM1-CNRS 5290-IRD 224, IRD Montpellier, Montpellier, France; Laboratoire d’Écologie et Biologie évolutive, Département de Biologie Animale, Faculté des Sciences et Techniques, Université Cheikh Anta Diop de Dakar, BP 5005 Dakar, Senegal

**Keywords:** *Plasmodium*, *Pan troglodytes*, Chimpanzee, Symptom, Malaria, Anaemia, Hyperthermia, Fever

## Abstract

Although *Plasmodium* infections have never been clearly associated with symptoms in non-human primates, the question of the pathogenicity of *Plasmodium* parasites in non-human primates still remains unanswered. A young chimpanzee, followed before and after release to a sanctuary, in a semi-free ranging enclosure located in an equatorial forest, showed fever and strong anaemia associated with a high *Plasmodium reichenowi* infection, shortly after release. The animal recovered from anaemia after several months despite recurrent infection with other *Plasmodium* species. This may be the first description of malaria-like symptoms in a chimpanzee infected with *Plasmodium*.

## Background

**Fig. 1:**
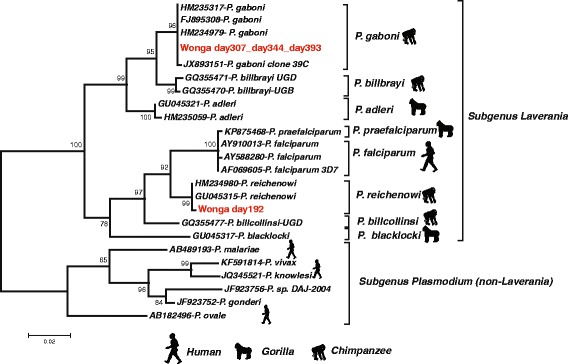
Phylogenetic relationships among primate *Plasmodium* based on *cytochrome b* sequences (680 bp). Sequences obtained for Wonga at the different checkups are included in red. The sequences reported in this study were deposited in Genbank under the following accession numbers: KR350681 to KR350683

Non-human primates and particularly great apes are natural hosts of various *Plasmodium* parasites [1, 2], including species very closely related to human parasites, like *Plasmodium falciparum* (the most virulent agent of human malaria), *Plasmodium malariae*, *Plasmodium ovale* and *Plasmodium vivax* (Fig. 1) [3, 4]. Among the species related to *P. falciparum* and classified into the subgenus *Laverania*, three species were shown to infect only chimpanzees (*Plasmodium gaboni*, *Plasmodium billcollinsi* and *Plasmodium reichenowi*) and three only gorillas (*Plasmodium adleri, Plasmodium blacklocki* and *Plasmodium praefalciparum).* For the species related to *P. malariae, P. ovale* and *P. vivax* and classified in the subgenus *Plasmodium,* they were all shown to infect both chimpanzees and gorillas.

Despite their genetic proximity with human parasite species especially *P. falciparum*, it is still unclear whether ape *Plasmodium* are virulent, that is whether great apes express disease or not when they are infected [5]. A case of a fatal *Plasmodium* infection in a chimpanzee was previously described [6], as well as in a gorilla [7], but the exact nature of the parasite involved was never determined with molecular tools, thus leaving the possibility of an infection with a human parasite (*P. falciparum* in particular). In addition, other pathogens could have caused these deaths and no special efforts were made to reject these possibilities. In contrast, other authors showed that the presence of *Plasmodium* in African great apes was not correlated with any increase of temperature or any other symptoms [1] or that the haematologic and biochemical values, as well as the majority of immunologic parameters, remained unaltered in chimpanzees infected with *P. falciparum* [8].

Some researchers noticed malaria-like symptoms responding to anti-malaria treatment in orangutans but without clear evidence of the presence of the pathogen [9]. In non-human primates other than great apes, studies showed that experimental infections of rhesus macaques with *Plasmodium knowlesi*, *Plasmodium coatneyi*, and less often *Plasmodium cynomolgi*, may be characterized by jaundice, anorexia, listlessness, fever, anaemia, and splenomegaly in spleen-intact animals [10]. Moreover, *P. knowlesi*-infected baboons expressed either acute infection with multiple system organ dysfunctions and cerebral involvement, or a chronic infection with spleen enlargement [11].

Therefore, no evidence of symptoms was ever clearly associated with natural *Plasmodium* infection in non-human primates, especially in great apes. This case present what could be the first description of symptoms associated to a clearly identified natural *Plasmodium* infection in a chimpanzee.

## Case presentation

### History

A 6-year-old female chimpanzee (*Pan troglodytes troglodytes*), named Wonga, was brought to the Centre International de Recherches Médicales de Franceville (CIRMF) after being confiscated from illegal owners in Libreville, Gabon. She was captured by the owners after they killed her mother and was kept as a pet for most of her life, living inside the owners’ house in an urban environment. She spent her quarantine at CIRMF and cleared out after 90 days. She was then transferred to the sanctuary “Parc de la Lékédi”, Bakoumba (Haut-Ogooué, Gabon).

The sanctuary “Parc de la Lékédi”, Bakoumba, holds various primates species, including gorillas, chimpanzees and small monkeys, all bushmeat-poaching-issued orphans, which have been confiscated by the Gabonese Government, gone through a quarantine period at the Centre International de Recherches Médicales de Franceville (CIRMF) and finally released into semi-free ranging enclosures in the sanctuary. Wonga was released into a group of 10 chimpanzees, ranging from 3 to 12 year-old, sharing a 7-ha-semi-free ranging enclosure in dense equatorial forest. The chimpanzees are left in the enclosure nights and days, and sleep in the trees. Food supplementation is offered every day, around 10 am and 2 pm, and consists of fruits and protein cakes. During feeding, keepers monitor animals’ global health on site. Contacts with humans are extremely limited, to annual medical check-ups or health-care-needed anesthesia. Chimpanzees are not followed during the day and evolve freely into the enclosure.

Three and a half months after Wonga’s release into the group, routine medical annual check-up was performed on all the chimpanzees of the group.

All procedures in this study involving animals were approved by the Government of the Republic of Gabon. This study complied with the relevant national guidelines and with the IUCN Policy Statement on Research Involving Species at Risk of Extinction.

### Clinical examinations

**Fig. 2:**
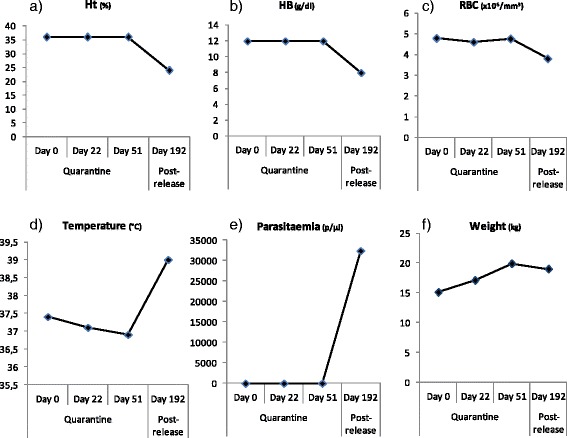
Evolution of haematology parameters, temperature, parasitaemia and weight between quarantine (days 0, 22 and 51) and first post-release check-up (day 192). **a** Haematocrit (%); (**b**) Haemoglobin levels (g/dl); (**c**) Red blood cell count (×10^6^/mm^3^); (**d**) Temperature (°C); (**e**) Parasitaemia (parasites/μl of blood); (**f**) Weight (kg). Ht: Haematocrit; HB: Haemoglobin; RBC: Red blood cells

During her quarantine, three clinical check-ups were performed on days 0, 22 and 51, following CIRMF quarantine protocol. For every medical check-up, the animal was anaesthetized with ketamine (10 mg/kg) via intramuscular injection. Once asleep, blood was collected from femoral vena into EDTA and dry tubes, on which pathogen screenings were performed. Wonga expressed no clinical symptoms of any disease or infection. Her temperature was stable over these check-ups (mean = 37.1 °C; SD = 0.25166). She constantly gained weight over the three check-ups, which is expected from an individual recently confiscated and poorly nourished upon arrival at CIRMF (Fig. 2f).

**Fig. 3:**
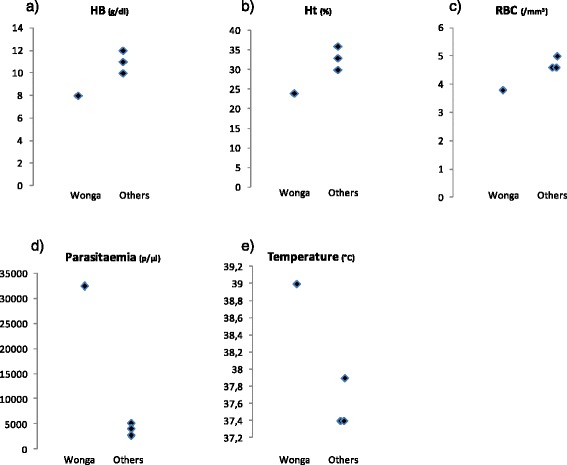
Blood results, temperature and parasitaemia comparison between Wonga and other chimpanzees on day 192. **a** Haemoglobin levels (g/dl); (**b**) Haematocrit (%); (**c**) Red blood cell count (×10^6^/mm^3^); (**d**) Parasitaemia (parasites/μl of blood); (**e**) Temperature (°C). Ht: Haematocrit; HB: Haemoglobin; RBC: Red blood cells

Three and a half months after her transfer to the Parc de la Lékédi, on day 192, annual check-up of all chimpanzees of the group was routinely performed. Four chimpanzees (including this animal) were sampled on the same day. They were anaesthetized with ketamine (10 mg/kg) via intramuscular injection. The veterinarians noticed that Wonga was less active, calmer and sleepier than usual and than the other chimpanzees. Her physical examination revealed no abnormalities, except that her rectal temperature was high (39 °C), unlike her previous temperatures observed during quarantine (Fig. 2d) and unlike the three other chimpanzees examined the same day (mean = 37.56 °C, SD = 0.28868) (Fig. 3e). Veterinarians also noticed that Wonga did not gain any weight since her quarantine (Fig. 2f).

### Laboratory tests

#### The chimpanzee Wonga

##### Blood count results

For each check-up during quarantine and after release, blood counts (serum chemistry and haematology) were performed. Serum chemistry results showed no strong abnormalities all along check-ups (Table 1) [12]. During her quarantine at CIRMF, Wonga was sampled three times. Her haematologic results were within or slightly below normal ranges for juvenile female chimpanzees (Table 2) [12].

**Table 1:** Wonga serum chemistry results

	Parameters	Alb	Tot	Creat	Urea	GGT	AST	ALT	ALP
		(g/dL)	Prot (g/dL)	(mg/dL)	(mg/dL)	(U/L)	(U/L)	(U/L)	(UI/L)
	(Normal ranges)	(3.1–4.8)	(6.2–8.1)	(0.4–1)	(4.6–19.9)	(7.3–36.4)	(7.9–33.0)	(18.6–61.1)	(159.1–1080.1)
Quarantine	Day 0	N/A	**8.5**	0.87	11.5	**4.92**	16	**18**	**1.5**
	Day 22	N/A	**8.8**	0.78	5.88	10	19	21	**40**
	Day 51	3.8	**6.0**	0.9	**2.8**	N/A	13	**15**	538
Post-release	Day 192	**3**	7.6	0.78	5.6	14	**40**	23	288
	Day 307	**2.9**	8.1	0.64	**1.12**	22	24	19	324
	Day 344	3.4	N/A	0.62	N/A	27	24	**13**	270
	Day 393	3.4	**8.4**	0.62	11.54	16	19	20	374

*Alb* albumin, *Tot Prot* total protein, *Creat* creatinine, *Urea* urea nitrogen, *GGT* γ-glutamyltransferase, *AST* aspartate transaminase, *ALT* alanine transaminase, *ALP* alkaline phosphatase

Bold values: values out of normal ranges

**Table 2:** Wonga hematological results

	Parameters	RBC	HB	Ht	Plt	MCV	MCH	MCHC
		(×10^6^/mm^3^)	(g/dl)	(%)	(/mm^3^)	(μm^3^)	(pg/ml)	(g/dl)
	(Normal ranges)	(4.0–5.9)	(11.2–15)	(34.5–45.9)	(171,900–442,000)	(74.0–87.9)	(23.7–28.9)	(30.6–34.5)
Quarantine	Day 0	4.79	12	36	371,000	**70**	24	35
	Day 22	4.6	12	36	431,000	**69**	25	36
	Day 51	4.76	12	36	365,000	**69**	25	37
Post-release	Day 192	**3.8**	**8**	**24**	266,000	**68**	**20**	32

*RBC* red blood cells, *HB* hemoglobin, *Ht* hematocrit, *Plt* platelets, *MCV* mean cell volume, *MCH* mean cell hemoglobin, *MCHC* mean cell hemoglobin concentration

Bold values: values out of normal ranges

During her first post-release check-up (day 192), correlated with her fever, her haematological results showed anaemia, with low haematocrit, low haemoglobin level and low red blood cell count (Table 2 and Fig. 2).

##### *Plasmodium* infection detection

Along with blood counts, Wonga was tested for *Plasmodium* infections during quarantine and after release using two distinct diagnostic tests (thick blood smears and *cytochrome b* PCR/sequencing) [13]. The three samples tested during quarantine showed no *Plasmodium* infection (Fig. 2e).

**Fig. 4:**
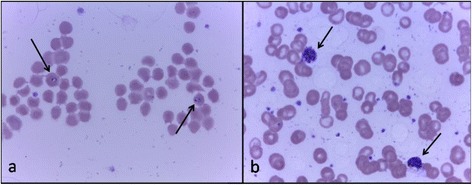
Wonga’s *Plasmodium sp.* forms on day 192. **a** Ring form; (**b**) Mature schizonts. Giemsa stain, 100 × magnification. Arrows point towards the different forms

On day 192, correlated with her fever and anaemia, she was tested positive for *P. reichenowi* (Fig. 1), with a high parasitaemia (32,472 parasites/μl of blood) (Fig. 2e). Parasitaemia was determined by counting the number of parasites infecting red blood cells, over a population of 100 white blood cells. Then, knowing the white blood cell count at the same date, the density of *Plasmodium* parasites per microlitre of blood was calculated. Several forms were observed including ring stages and mature forms like schizonts (Fig. 4), indicating that the parasite was clearly developing and could be the origin of the hyperthermia and of the anaemia.

##### Other infections

During quarantine and for the three samples, the chimpanzee was screened for several pathogens. Simian Immunodeficiency Virus (SIV, peptides set), Simian T-Cell Leukaemia Virus (STLV, peptides set), hepatitis B (HBV, serology), hepatitis C (HCV, serology), filariosis (leucoconcentration), bacterial infections (bacterial cultures performed on nasal and anal swabs), tuberculosis (intradermal palpebral test) and gastro-intestinal parasites (Bailenger and flotation techniques) were all tested negative. Nevertheless, anti gastrointestinal parasites drugs were preventively administered.

On day 192, filariosis (leucoconcentration), bacterial infections (bacterial cultures performed on nasal and anal swabs), gastro-intestinal parasites (Bailenger and flotation techniques) and tuberculosis (intradermal palpebral test) were checked negative. Because the other chimpanzees of the group are non-infected with SIV, STLV, HBV and HCV and because animals do not have any contacts with wild chimpanzees, these pathogens were not checked on Wonga on day 192.

#### Comparison to other chimpanzees

Three other chimpanzees, two juvenile females (5 and 6 years old) and one adolescent male (9 years old), were sampled on day 192. Their haemoglobin levels (HB), haematocrit (Ht), and red blood cell counts (RBC) were within or slightly lower than chimpanzees’ normal haematological ranges (for juvenile females: HB = 11.2–15 g/dl; Ht = 34.5–45.9 %; RBC = 4.0–5.9 × 10^6^/mm^3^; for adolescent males: HB = 12.4–16.4 g/dl; Ht = 37.9–49.7 %; RBC = 4.5–6.1 × 10^6^/mm^3^) [12]. They did not show either anaemia or hyperthermia, unlike Wonga (Fig. 3).

All chimpanzees of the group were infected with *Plasmodium* (two of them with *P. reichenowi* and one with *P. malariae)*, but with a much lower parasitaemia than Wonga (2720, 4048 and 5130 parasites/μl) (Fig. 3d).

### Evolution

**Fig. 5:**
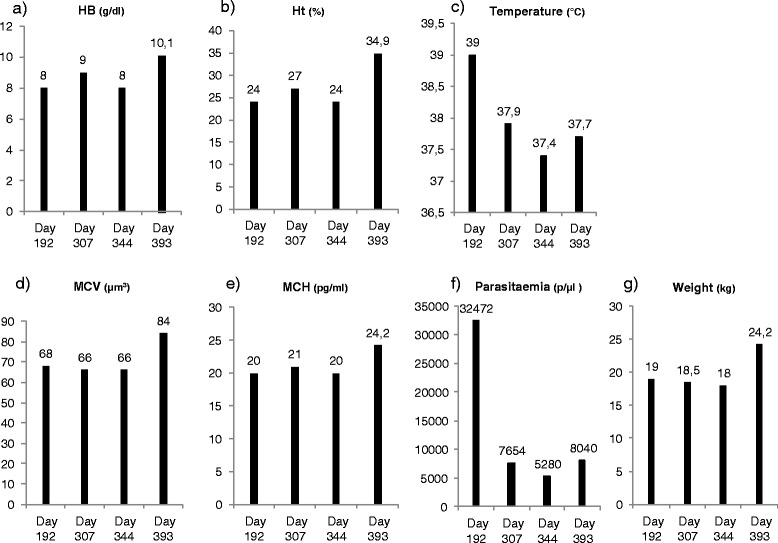
Evolution of Wonga’s haematological parameters, temperature, parasitaemia and weight during post release check-ups (days 192, 307, 344, and 393). **a** Haemoglobin levels (g/dl); (**b**) Haematocrit (%); (**c**) Temperature (°C); (**d**) Mean cell volume (μm^3^); (**e**) Mean cell haemoglobin (pg/ml); (**f**) Parasitaemia (parasites/μl of blood); (**g**) Weight (kg). Ht: Haematocrit; HB: Haemoglobin; MCV: Mean cell volume; MCH: Mean cell haemoglobin

Wonga stopped showing any abnormal behaviour some days after day 192. On days 307, 344 and 393, she was anaesthetized again for vaccination purposes. The veterinarian took advantage of the anesthesia needed for vaccinations to perform a full clinical examination and to sample the animal. Her temperatures had decreased back to normal (37.9, 37.4 and 37.7 °C) (Fig. 5c).

*Plasmodium* sequencing showed that she was now infected with *P. gaboni* (Fig. 1). Her parasitaemia was still high but strongly decreased compared to day 192 (Fig. 5f).

On days 307 and 344, her haematological results still showed strong anaemia (Fig. 5). RBC count was back within normal range, but Hb level, Ht, mean cell volume (MCV) and mean cell haemoglobin (MCH) were low, signing a chronic microcytic and hypochromic anaemia. On day 393, Wonga’s laboratory results showed that she recovered from her anaemia, with much higher Ht, Hb level, MCV and MCH (Fig. 5).

Follow-up on Wonga’s weight was also performed (Fig. 5g); after her release to the sanctuary, she was barely maintaining her weight until day 344. Then, between day 344 and day 393, as she recovered from her anaemia, she spectacularly gained weight in a short period of time.

On day 307, she was tested negative for gastrointestinal infections; however, anti gastrointestinal parasite drug was preventively administered on that day. On days 344 and 393, she was again tested negative for gastrointestinal parasites infections.

## Discussion

### The case

Some weeks after her release in a dense tropical forest, Wonga, a 6-year-old female chimpanzee, presented a fever associated with anaemia, characterized by a very low Ht, very low Hb level and a low RBC count, as well as no weight gain since her quarantine. These clinical and laboratory findings were associated with a *Plasmodium* infection at high parasitaemia. All her symptoms (especially high parasitaemia and strong anaemia) are characteristics of a strong malaria attack in a naïve individual. Indeed, Wonga was kept for most of her life inside a house in an urban environment and may never have been confronted to a *Plasmodium* infection. Moreover, Wonga was not infected during quarantine, which lasted 3 months. *Plasmodium*-acquired immunity is believed not to last for a long period of time [14], thus Wonga may be considered naïve regarding *Plasmodium* infection. Given her history, the symptoms Wonga expressed may thus be due to the reaction of a naïve animal experiencing its first *Plasmodium* infection. On the opposite, the other chimpanzees sampled, which have been followed for the past 2 years and have constantly been tested positive for *Plasmodium*, are definitively not naïve regarding *Plasmodium* infection. On day 192, Wonga expressed two features that are seen in humans experiencing their first *Plasmodium* infection: high parasitaemia and severe disease, including severe anaemia. Malaria-naïve patients have significantly higher risk to develop a peak parasitaemia compared to patients born and resident in malaria-endemic countries of Africa [15]. Wonga, a malaria-naïve chimpanzee, did express a peak parasitaemia compared to other chimpanzees resident in the malaria-endemic sanctuary. High levels of parasitaemia can result in substantial destruction of red blood cells, causing a severe anaemia, particularly in non-immune individuals [16]. However, even if *Plasmodium-*induced haemolysis contributes to a reduction in haemoglobin levels, low haemoglobin levels in anaemic children may also be explained by ineffective erythropoiesis [16]. Studies demonstrated that patients experiencing acute *falciparum* malaria show bone marrow damage, ineffective erythropoiesis and a reduced rate of erythropoietic proliferation [17]. Therefore, high parasitaemia and ineffective erythropoiesis may explain Wonga’s anaemia. Severe anaemia is also a feature more often expressed by non-immune individuals infected with *Plasmodium;* it has been shown that non-immune tourists going to *Plasmodium-*endemic areas are at greater risk to develop severe disease than semi-immune residents, who seem at lower risk for severe disease [18]. This is also true for children experiencing their first malaria infection: severe disease with rapid progression to death occurs in young children without acquired immunity [19]. Most of the mortality associated with *P. falciparum* infection occurs in immune-naïve African children under 5 years of age. Symptoms of severe *P. falciparum* malaria include hyperparasitaemia and severe malarial anaemia [16]. Therefore, Wonga’s non-acquired immunity to *Plasmodium* may explain the hyperthermia and anaemia she experienced, while semi-immune chimpanzees, living in the area for years, did not show any symptoms. Unfortunately, no other *Plasmodium*-naïve chimpanzees recently introduced in the area was anaesthetized soon after release, thus allowing the same study as Wonga’s. Anaesthesia and sampling are necessary to detect anaemia and hyperthermia, which may explain why malaria-like symptoms were never detected on the other chimpanzees previously released. Given the unique aspect of the case observed, further case studies will thus be necessary to assess whether treatment should be administered.

No difference on alimentation, stress or weather could have biased these results. All individuals compared in this study were sampled the same day, with the same technique and conditions, and live in the same enclosure. None of them suffered malnutrition and all presented a good body condition. No difference in capture, anaesthesia or amount of stress could have been the origin of such a raise in temperature. Wonga was also tested for other diseases which could have been responsible for the fever (for example, bacterial infections), and for the anaemia (for instance, gastrointestinal parasites such as *Ancylostoma sp.)*.

### Evolution of the case

After several months (day 307), *P. reichenowi* had disappeared from Wonga’s blood, but another malaria infection with *P. gaboni* was observed, associated to a lower parasitaemia. On days 307 and 344, Wonga did not show any hyperthermia but still had microcytic and hypochromic anaemia. During the same period, she did not gain any weight, confirming that her global health was still not optimum. Gastrointestinal parasites infections which could be responsible for these symptoms were again ruled out.

The clearance of *P. reichenowi* from Wonga could have been due to the development of an immunity of the chimpanzee against this parasite species. The existence of such immunity against chimpanzee parasite was recently indirectly evidenced in natural ape populations by showing that prevalence of infection was lower in older individuals [20]. Regarding *P. gaboni*, infection was acquired by Wonga any time between day 192 and day 307. Whether this infection induced similar symptoms cannot be told. Further studies should be done to determine if all ape *Plasmodium* species induce similar symptoms or not. It is nevertheless likely that this infection, even at a low parasitaemia, was partly responsible for the maintenance of the anaemia.

Finally, between day 344 and day 393, after several months without any symptoms other than anaemia and no weight gain, the chimpanzee recovered from her haematological condition, recovered usual haemoglobin levels, and resumed gaining weight. Such recovery could be explained by a progressive accommodation of Wonga’s physiology to the recurrent *Plasmodium* infections [21], as observed in humans [22].

## Conclusions

Wonga expressed hyperthermia and strong anaemia correlated with a high parasitaemia *Plasmodium* infection. Other possible causes of the symptoms observed were ruled out. Then, after several months, and despite recurrent infection with other *Plasmodium* species, the animal gradually recovered from anaemia. Symptoms such as fever and anaemia were consistent with a strong *Plasmodium* infection, and showed first evidence of malaria-like symptoms in a likely naïve-chimpanzee infected with *Plasmodium.*
